# Efficacy of Inebilizumab in N‐MOmentum Trial Participants With or Without Prior Immunosuppressants

**DOI:** 10.1002/acn3.70426

**Published:** 2026-05-12

**Authors:** Bruce A. C. Cree, Beatrice Suero, Sarah Walsh, Romain Marignier, John W. Lindsey, Ho Jin Kim, Dewei She, Daniel Cimbora, Dustin Cavida, Friedemann Paul

**Affiliations:** ^1^ UCSF Weill Institute for Neurosciences, Department of Neurology University of California San Francisco California USA; ^2^ Eversana Burlington Ontario Canada; ^3^ Service de Neurologie, Sclérose en Plaques, Pathologies de la Myéline et Neuroinflammation Hôpital Neurologique Pierre Wertheimer, Hospices Civils de Lyon Lyon France; ^4^ Division of Multiple Sclerosis and Neuroimmunology University of Texas Health Science Center at Houston Houston Texas USA; ^5^ Department of Neurology Research Institute and Hospital of National Cancer Center Goyang Republic of Korea; ^6^ Amgen Inc. California USA; ^7^ Experimental and Clinical Research Center Max Delbrueck Center for Molecular Medicine and Charité – Universitätsmedizin Berlin, Corporate Member of Freie Universität Berlin and Humboldt‐Universität Zu Berlin Berlin Germany

**Keywords:** aquaporin‐4 (AQP4), inebilizumab, long‐term management, neuromyelitis optica spectrum disorder (NMOSD), prior immunosuppression

## Abstract

This post hoc analysis examined the impact of prior immunosuppressants on the long‐term efficacy and safety of inebilizumab, a cluster of differentiation 19^+^ B‐cell–depleting monoclonal antibody, in participants with aquaporin‐4–seropositive neuromyelitis optica spectrum disorder from the N‐MOmentum trial (NTC02200770). Inebilizumab treatment resulted in a sustained low annualized attack rate relative to the pretrial annualized attack rate and a high probability of remaining attack‐free for up to 4 years among participants with and without prior immunosuppressant use. Based on modeling data, inebilizumab had greater long‐term efficacy than historical immunosuppressants. Inebilizumab was well tolerated regardless of prior immunosuppressant use.

## 1 Introduction

Commonly prescribed, off‐label treatments for neuromyelitis optica spectrum disorder (NMOSD) include immunosuppressants (ISTs), such as azathioprine (AZA) and mycophenolate mofetil (MMF) [1]. Oral ISTs are associated with greater frequency of relapse occurrence and a shorter time to relapse following initiation of treatment vs. approved monoclonal antibodies [2, 3, 4]. Additionally, oral ISTs are associated with gastrointestinal and hematologic side effects and a high frequency of infections [5].

The efficacy and safety of inebilizumab (INEB)—a humanized, affinity‐optimized, glycoengineered monoclonal antibody targeting cluster of differentiation 19^+^ (CD19^+^) B cells [6]—in NMOSD were assessed in the randomized, placebo‐controlled N‐MOmentum trial that led to regulatory approval of INEB in aquaporin‐4‐seropositive (AQP4^+^) NMOSD [6, 7]. Many participants in N‐MOmentum had a history of IST use, which could potentially impact the efficacy and safety of INEB [8]. Here, the long‐term efficacy and safety of INEB in participants with and without prior IST treatment from N‐MOmentum is reported. As the randomized, controlled period (RCP) of N‐MOmentum lasted approximately 28 weeks, modeled data were used to assess the long‐term efficacy of INEB compared to synthetic historical placebo and IST comparator groups.

## 2 Methods

### 2.1 Trial Design and Analysis

The trial design for N‐MOmentum (NCT02200770) was previously described; brief details are provided in Figure S1 and the Supporting Information [8, 9]. Participants who received at least one INEB treatment at any point in the RCP or open‐label period (OLP) of N‐MOmentum are referred to as the any INEB population. This post hoc exploratory analysis included participants with AQP4^+^ NMOSD with prior AZA, MMF, and/or methotrexate treatment or participants who were IST naïve. The IST‐naïve group included some participants who had prior treatment with less commonly used ISTs. Only participants with AQP4^+^ NMOSD were assessed in this analysis given the approved use of inebilizumab in these patients. ISTs to prevent or treat NMOSD relapses were allowed prior to dosing on day 1 but prohibited during N‐MOmentum. Briefly, exclusion criteria included estimated glomerular filtration rate < 60 mL/min; pregnant females; alcohol, drug, or chemical abuse history < 1 year prior to randomization; major surgery; uncontrolled hypertension; clinically significant viral or bacterial infection; and failed screening for laboratory parameters (e.g., liver enzymes and blood cells counts) [8, 9]. Participants receiving rituximab or B‐cell depletion within the 6 months prior to screening were also excluded [7, 8]. Endpoints included the time to onset of an adjudicated NMOSD attack, annualized attack rate (AAR), the number of NMOSD‐related inpatient hospitalizations, and changes from baseline in neurological disability, measured by the Expanded Disability Status Scale (EDSS). All *p* values reported from this analysis are nominal, and statistical methods related to this analysis are described in the Supporting Information.

N‐MOmentum was conducted in accordance with the International Conference on Harmonization Good Clinical Practice Guidelines and principles of the Declaration of Helsinki. An ethics committee or institutional review board at each trial site approved the protocol. Written informed consent was obtained from all participants prior to performing any trial examinations or procedures [8, 9].

### 2.2 Historical Comparator Groups

In the absence of long‐term placebo or IST comparator data in the OLP, historical data from patients treated with placebo or those treated with AZA or other broad‐spectrum ISTs (AZA/IST) in published NMOSD studies were used to evaluate the long‐term comparative efficacy of INEB (see Suppporting Information). Attack risk was assessed using parametric and flexible survival (spline) models. Model selection was guided by testing Cox proportional hazards and accelerated failure time assumptions, and further informed by Akaike or Bayesian information criterion, visual fit, estimated attack‐free survival at 4 years, and clinical validation. A time‐varying treatment‐effect spline model with two internal knots and a normal linear predictor was determined as the best‐fitting model and was applied to INEB data from the OLP and the historical comparator groups. Pseudo‐individual patient‐level data were reconstructed from the published Kaplan–Meier curves from the historical comparator groups. The spline model was fit to Kaplan–Meier data to assess comparative efficacy between the three groups. The spline model is described in further detail in the Supporting Information. The attack‐free survival probability was similar between the historical placebo group and the placebo arm during the RCP of N‐MOmentum, demonstrating the appropriateness of using the historical placebo comparator group in this analysis [8].

## 3 Results

### 3.1 Baseline Characteristics

Of the 231 participants randomized in N‐MOmentum, 213 were AQP4^+^ [8]. In the OLP, there were 201 AQP4^+^ participants [8]. This analysis included 202/213 AQP4^+^ participants from the RCP and 197/201 AQP4^+^ participants from the OLP [8]. In both the RCP and the OLP, approximately 50% of participants had prior IST treatment and 50% were IST naïve. The mean age was approximately 43 years, and most participants were female (prior IST, 87/96 [90.6%]; IST naïve, 103/106 [97.2%]) and White (prior IST, 44/96 [45.8%]; IST naïve, 62/106 [58.9]). More participants of Asian descent (prior IST, 33/96 [34.4%]; IST naïve, 10/106 [9.4%]) had prior IST use than no prior IST use, whereas more participants in the White, Black or African American (prior IST, 2/96 [2.1%]; IST naïve, 14/106 [13.2%]), and American Indian or Alaskan Native (prior IST, 3/96 [3.1%]; IST naïve, 13/106 [12.3%]) populations were IST naïve (Table 1).

**TABLE 1:** Baseline demographics and clinical characteristics by prior IST use.

	Prior IST treatment^a^(*n* = 96)	IST naïve^b^(*n* = 106)
Age, years, mean ± SD	43.3 ± 12.0	42.9 ± 12.8
Sex, female, *n* (%)	87 (90.6)	103 (97.2)
Disease duration, years, mean ± SD	3.45 ± 3.71	1.76 ± 3.04
Time to first INEB administration, years, median (range)	4.68 (0.38–27.44)	1.58 (0.11–26.28)
Number of MRI lesions, mean ± SD	1.02 ± 1.10	1.17 ± 1.11
EDSS score, median (range)	3.50 (0.0–8.0)	3.50 (0.0–8.0)
BMI, kg/m^2^, mean ± SD	25.49 ± 5.52	26.13 ± 6.45
Race, *n* (%)		
American Indian or Alaskan Native	3 (3.1)	13 (12.3)
Asian	33 (34.4)	10 (9.4)
Black or African American	2 (2.1)	14 (13.2)
White	44 (45.8)	62 (58.9)
Multiple categories checked	0	1 (0.9)
Other	14 (14.6)	6 (5.7)

Abbreviations: AZA, azathioprine; BMI, body mass index; EDSS, Expanded Disability Status Scale; INEB, inebilizumab; IST, immunosuppressant; MMF, mycophenolate mofetil; MRI, magnetic resonance imaging; SD, standard deviation.

^a^ Prior IST treatment included AZA, MMF, and/or methotrexate.

^b^ The IST‐naïve group includes some participants who had prior treatment with less commonly used ISTs.

### 3.2 Efficacy in the RCP

During the RCP, fewer participants who received INEB had an adjudicated attack vs. those who received placebo, both in participants with prior IST treatment (hazard ratio [HR; 95% confidence interval (CI)], 0.21 [0.09, 0.48]) and in those who were IST naïve (HR [95% CI], 0.23 [0.09, 0.59]; Table 2). A smaller proportion of participants who received INEB had NMOSD‐related inpatient hospitalizations vs. participants who received placebo, regardless of prior IST history (Table 2). During the RCP, the difference in the proportion of participants with worsening EDSS scores between those receiving INEB vs. placebo was statistically significant among both participants with prior IST treatment (19.2% vs. 43.5%; *p* < 0.05 vs. placebo) and those who were IST naïve (13.6% vs. 28.0%; *p* < 0.05 vs. placebo; Table 2).

**TABLE 2:** Adjudicated attacks, NMOSD‐related inpatient hospitalization rates, and EDSS score worsening during the RCP.

	AQP4^+^			
	Prior IST treatment^a^		IST naïve^b^	
	RCP PBO (*n* = 23)	RCP INEB (*n* = 73)	RCP PBO (*n* = 25)	RCP INEB (*n* = 81)
Number of participants with an attack, *n* (%)	13 (56.5)	10 (13.7)	9 (36.0)	8 (9.9)
HR (95% CI)	—	0.21 (0.09, 0.48)	—	0.23 (0.09, 0.59)
Participants with NMOSD‐related hospitalizations, *n* (%)^c^	4 (17.4)	3 (4.1)	3 (12.0)	6 (7.4)
Min, max	1, 2	1, 1	1, 1	1, 1
Rate ratio (95% CI)	—	0.14 (0.03, 0.68)	—	0.62 (0.15, 2.47)
*p* value^d^	—	0.01	—	0.50
EDSS score worsening				
Participants with EDSS worsening, *n* (%)	10 (43.5)	14 (19.2)	7 (28.0)	11 (13.6)
OR (95% CI)	—	0.30 (0.11, 0.83)	—	0.42 (0.14, 1.28)

Abbreviations: AQP4^+^, aquaporin‐4–seropositive; AZA, azathioprine; CI, confidence interval; EDSS, Expanded Disability Status Scale; HR, hazard ratio; INEB, inebilizumab; IST, immunosuppressant; MMF, mycophenolate mofetil; max, maximum; min, minimum; NMOSD, neuromyelitis optica spectrum disorder; PBO, placebo; OR, odds ratio; RCP, randomized, controlled period.

^a^ Prior IST treatment included AZA, MMF, and/or methotrexate.

^b^ The IST‐naïve group includes some participants who had prior treatment with less commonly used ISTs.

^c^ The descriptive statistics are based on the participants who were hospitalized.

^d^ *p* values are nominal and were calculated for INEB vs. PBO during the RCP.

### 3.3 Long‐Term Efficacy

**FIGURE 1:**
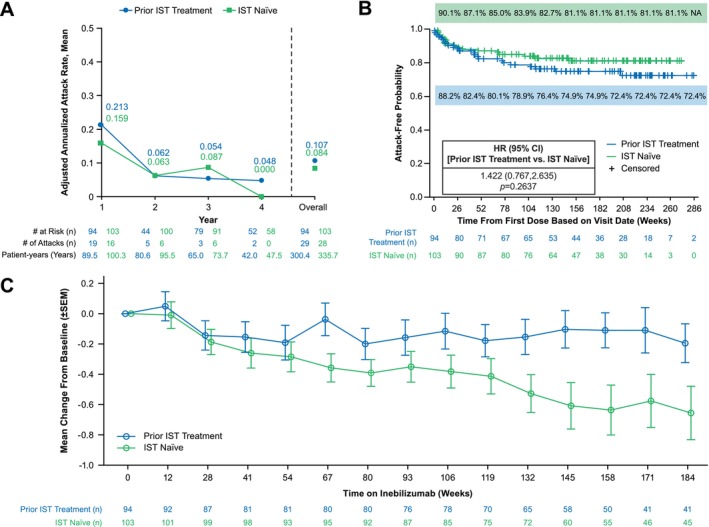
Long‐term efficacy of INEB in participants with or without prior IST treatment during the N‐MOmentum trial by (A) annualized attack rate, (B) probability of remaining attack‐free, and (C) mean change from baseline in EDSS scores. In panel A, for participants who received placebo during the RCP, data are only shown during the OLP after participants switched to INEB treatment. Prior IST treatment included AZA, MMF, and/or methotrexate. The IST‐naïve group includes some participants who had prior treatment with less commonly used ISTs. In panel B, participants who did not have an AC‐determined NMO/NMOSD attack before they completed or discontinued from the trial were censored at the time of the last trial visit. Prior IST treatment included AZA, MMF, and/or methotrexate. The IST‐naïve group includes some participants who had prior treatment with less commonly used ISTs. There were no participants in the IST‐naïve group at week 286. All *p* values reported are nominal. In panel C, prior IST treatment included AZA, MMF, and/or methotrexate. The IST‐naïve group includes some participants who had prior treatment with less commonly used ISTs. AC, adjudication committee; AZA, azathioprine; CI, confidence interval; EDSS, Expanded Disability Status Scale; HR, hazard ratio; INEB, inebilizumab; IST, immunosuppressant; MMF, mycophenolate mofetil; NA, not applicable; NMO, neuromyelitis optica; NMOSD, neuromyelitis optica spectrum disorder; OLP, open‐label period; RCP, randomized, controlled period; SEM, standard error of the mean.

For participants in the any INEB population, the adjusted overall AAR (95% CI) was 0.11 (0.07, 0.17) for participants with prior IST use vs. 0.08 (0.05, 0.14) for those without, demonstrating that the AAR with INEB is similar regardless of prior IST use (Figure 1A). There was a high probability of all participants treated with INEB remaining attack‐free up to week 286 of treatment (Figure 1B). Regardless of IST history, INEB treatment during the OLP resulted in improvements in EDSS scores among participants in the any INEB population (Figure 1C). Among participants who received INEB at any time during the trial, there were 40 neuromyelitis optica–related hospitalizations each for those with (*n* = 94) and without (*n* = 103) prior IST use (Table S1).

### 3.4 Long‐Term Efficacy of INEB vs. Historical AZA/IST and Placebo

**FIGURE 2:**
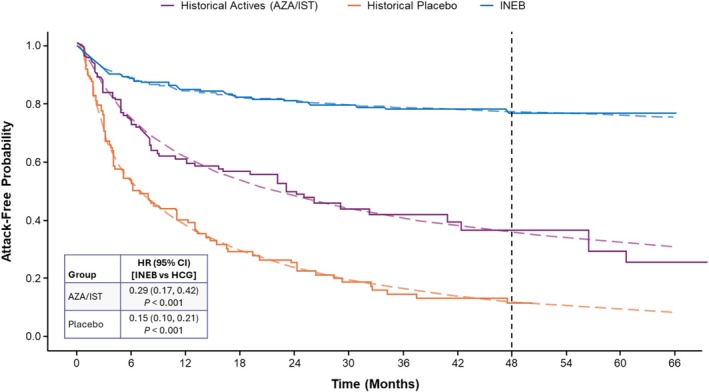
Modeled time to NMOSD attack: INEB vs. historical AZA/IST comparator and placebo groups. The time‐varying spline model uses two internal knots and a normal linear predictor. The solid lines represent the Kaplan–Meier data for historical AZA/IST and INEB in the OLP. The dashed lines represent the spline models that were fit to the Kaplan–Meier data. The vertical dashed line at 48 months highlights the attack‐free probability at 4 years, which was used to inform the spline model selection. The HRs (95% CI) are an estimate of the relative attack‐free probability over the entirety of the follow‐up period. All *p* values reported from this analysis are nominal AZA/IST, azathioprine or other broad‐spectrum immunosuppressants; CI, confidence interval; HCG, historical comparator group; HR, hazard ratio; INEB, inebilizumab; NMOSD, neuromyelitis optica spectrum disorder; OLP, open‐label period.

Historical data for AZA/IST and placebo from the published studies listed in Table S2 were used to assess the comparative efficacy of INEB in the OLP. The time to NMOSD attack was significantly lower with INEB vs. AZA/IST (HR [95% CI], 0.29 [0.17, 0.42]; *p* < 0.001) and with INEB vs. placebo (HR [95% CI], 0.15 [0.10, 0.21]; *p* < 0.001). Modeling data estimated a greater sustained attack‐free probability with INEB vs. AZA/IST and historical placebo (Figure 2). At 4 years, the estimated NMOSD attack‐free probability (95% CI) was 77% (71%, 83%) for INEB, 36% (27%, 46%) for AZA/IST, and 12% (7%, 20%) for placebo [9].

### 3.5 Safety

Treatment‐emergent adverse events (TEAEs) through the OLP are summarized in Table 3. AE frequencies were similar during the OLP for both groups. TEAEs related to INEB occurred more frequently in participants who were IST naïve vs. those with prior IST treatment. At least one AE of special interest was reported in both groups, with most events being related to infections. Infection rates were not increased in participants with vs. without prior IST use.

**TABLE 3:** Overall summary of TEAEs through the OLP.

	AQP4^+^	
	Prior IST treatment^a^ (*n* = 94)	IST naïve^b^ (*n* = 103)
≥ 1 AE	87 (92.6)	95 (92.2)
≥ 1 trial drug–related AE	29 (30.9)	48 (46.6)
≥ 1 SAE	15 (16.0)	23 (22.3)
≥ 1 trial drug–related SAE	4 (4.3)	6 (5.8)
≥ 1 AE leading to discontinuation of trial drug	4 (4.3)	2 (1.9)
Death	1 (1.1)	2 (1.9)
≥ 1 AESI	72 (76.6)	85 (82.5)
Infusion‐related reaction	11 (11.7)	13 (12.6)
Anaphylactic reaction	0	0
Hypersensitivity	1 (1.1)	1 (1.0)
Infections	68 (72.3)	79 (76.7)
Hepatic function abnormality	5 (5.3)	10 (9.7)
Cytopenia	3 (3.2)	9 (8.7)
Opportunistic infections^c^	1 (1.1)	1 (1.0)

*Note:* Participants were counted once for each category regardless of the number of events. All data are presented as *n* (%).

Abbreviations: AE, adverse event; AESI, adverse event of special interest; AQP4^+^, aquaporin‐4–eropositive; AZA, azathioprine; IST, immunosuppressant; MMF, mycophenolate mofetil; OLP, pen‐label period; SAE, serious adverse event; TEAE, treatment‐emergent adverse event.

^a^ Prior IST treatment included AZA, MMF, and/or methotrexate.

^b^ The IST‐naïve group includes some participants who had prior treatment with less commonly used ISTs.

^c^ The participant with prior IST treatment experienced a wound infection, and the participant who was IST naïve experienced a tooth infection.

## 4 Discussion

In N‐MOmentum, long‐term INEB treatment in participants with AQP4^+^ NMOSD resulted in similar low AARs and stabilized EDSS scores in participants with or without prior IST use. In the OLP, the AAR and probability of remaining attack‐free were similar for participants with and without previous IST use, suggesting that the long‐term efficacy of INEB is not influenced by prior IST use [8].

INEB was associated with reduced long‐term risk of an NMOSD attack vs. synthetic historical data for AZA/IST and placebo. In the absence of head‐to‐head clinical trials, this analysis suggests that the efficacy outcomes of INEB would be superior to AZA/IST. The increasing difference over time in the HR for the attack‐free probability between INEB and AZA/IST and between INEB and the synthetic historical placebo/untreated control suggests that the efficacy of INEB might increase with longer treatment duration.

NMOSD‐related hospitalizations with INEB treatment throughout the OLP were similar for participants with and without prior IST treatment. In the OLP, the nature and frequency of TEAEs were similar among participants treated with INEB, regardless of IST history, and the safety profile among both subgroups appeared to be similar to that of the overall trial population [8]. Switching from off‐label ISTs to immunotherapies likely requires overlapping treatment to avoid a risk of attack due to treatment discontinuation [9]. These results generally support INEB treatment for patients with NMOSD regardless of previous first‐line treatment with ISTs. Many providers use overlapping therapy with ISTs for up to 6 months when switching treatments [6, 10]. However, concomitant treatment with INEB and ISTs is not recommended in NMOSD as a regular treatment regimen [11].

Limitations of this post hoc analysis include the relatively small sample size for comparison of participants with and without prior IST exposures. Also, there is a risk of indication bias since randomization was not performed with respect to duration of IST history, and *p* values were not adjusted for multiple comparisons. An additional limitation is the use of synthetic historical comparator groups to estimate the long‐term comparative efficacy of INEB. The historical groups were synthesized by digitizing published Kaplan–Meier curves, which introduces potential bias and approximation error relative to analyses of individual patient‐level data. Additionally, differences in study populations and designs across studies may introduce confounding that cannot be fully mitigated through statistical modeling.

This analysis demonstrates that the efficacy of INEB is not influenced by prior IST use and that INEB has sustained long‐term efficacy in reducing the risk of NMOSD attack regardless of prior IST treatment. INEB was generally well tolerated in participants with and without prior IST use.

## Author Contributions

Conception/design: Bruce A. C. Cree, Romain Marignier, Ho Jin Kim, Dewei She, Daniel Cimbora, and Friedemann Paul. Data acquisition: Bruce A. C. Cree, John W. Lindsey, and Ho Jin Kim. Analysis/Interpretation: Bruce A. C. Cree, Beatrice Suero, and Sarah Walsh. Drafting/critical revision: Bruce A. C. Cree, Beatrice Suero, Sarah Walsh, Romain Marignier, John W. Lindsey, Ho Jin Kim, Dewei She, Daniel Cimbora, Dustin Cavida, and Friedemann Paul.

## Funding

This work was supported by Amgen.

## Conflicts of Interest

B.A.C. Cree reports personal compensation for consulting from Alexion Pharmaceuticals, Alumis, Avotres, Biogen, Boston Pharmaceuticals, EMD Serono, Hexal/Sandoz, Horizon Therapeutics, Immunic Therapeutics, Kyverna Therapeutics, Neuron23, Novartis, Sanofi, Siemens, and TG Therapeutics; received research support from Genentech and Kyverna Therapeutics; and is on an advisory board for Autobahn Therapeutics. B. Suero and S. Walsh report personal fees for consulting from Horizon Therapeutics (as part of EVERSANA). R. Marignier reports personal fees for consulting from Alexion Pharmaceuticals, Amgen Inc., Roche, and UCB. J.W. Lindsey reports personal compensation for speaking or consulting for Banner Life Sciences, Biogen, Celgene Corporation, EMD Serono, Genentech, Genzyme, Mapi Pharma, and TG Therapeutics; participated in clinical trials funded by Anokion, Atara Biotherapeutics, Biogen, EMD Serono, and Genentech; and has received research funding from Genentech and the National Multiple Sclerosis Society. H.J. Kim has received a grant from the Korea‐US Collaborative Research Project and the National Research Foundation of Korea; received research support from APRILBIO, Eisai, Good T Cells, and UCB; received consultancy/speaker fees from Alexion Pharmaceuticals, ALTOS Biologics, AstraZeneca, Biogen, Daewoong Pharmaceutical, Eisai, GC Pharma, Kaigene, MDimune, Merck, Mitsubishi Tanabe Pharma, Roche, Samsung Bioepis, and Sanofi; serves on a steering committee for MedImmune/Viela Bio; and is a coeditor for the *Multiple Sclerosis Journal* and an associate editor for the *Journal of Clinical Neurology*. D. She is a former employee of Amgen Inc. and a current employee of Acadia Pharmaceuticals and may hold stock in Amgen Inc. Cimbora and D. Cavida are employees of and stockholders in Amgen Inc. F. Paul has received research support, speaker honoraria, and travel reimbursement from Bayer, Biogen, the Guthy‐Jackson Charitable Foundation, Merck Serono, Novartis, Sanofi, and Teva Pharmaceutical; is supported by the German Research Council (DFG Exc 257) and the German Competence Network for Multiple Sclerosis; and serves on the steering committee of the OCTIMS study, sponsored by Novartis.

## Supporting information


**Figure S1:** N‐MOmentum trial design.
**Table S1:** NMO‐related inpatient hospitalization rates among participants treated with INEB during the N‐MOmentum trial.
**Table S2:** Summary of studies informing the HCGs.

## Data Availability

All relevant data from this analysis are included in the article and Supporting Information. Qualified researchers may request data from Amgen clinical studies. Complete details are available at the following link: https://www.amgen.com/science/clinical‐trials/clinical‐data‐transparency‐practices/clinical‐trial‐data‐sharing‐request.
