# Supplementary material for: Efficacy of Inebilizumab in N‐MOmentum Trial Participants With or Without Prior Immunosuppressants
Source: Ann Clin Transl Neurol. 2026 May 12:10.1002/acn3.70426. Online ahead of print. doi: 10.1002/acn3.70426 (PMC13394700; doi:10.1002/acn3.70426)
Supplement: Supplementary file 1 — Figure S1: N‐MOmentum trial design. Table S1: NMO‐related inpatient hospitalization rates among participants treated with INEB during the N‐MOmentum trial. Table S2: Summary of studies informing the HCGs. [file ACN3-9999-0-s001.docx]

**SUPPLEMENTAL APPENDIX**

**METHODS**

**Trial Design and Analysis**

N-MOmentum was a double-blind, placebo-controlled, randomized, phase 2/3 clinical trial that assessed the efficacy and safety of inebilizumab (INEB) in adults with neuromyelitis optica spectrum disorder (NMOSD). ^1,2^ Participants with a history of immunosuppressants (ISTs) were permitted to enroll in N-MOmentum; those with rituximab or B-cell depletion within the 6 months prior to screening were excluded. ^1,2^ Eligible participants were enrolled in the 28-week randomized, controlled period (RCP), where they received INEB (300 mg) or placebo intravenously on days 1 and 15. Participants who continued into the optional open-label period (OLP) received INEB on OLP day 1 (and OLP day 15 for participants who received placebo in the RCP) and then every 26 weeks for ≥2 years (**Supplementary Figure 1**). Participants could switch to the OLP whenever they experienced the first attack; therefore, the RCP duration varied among participants.

**Statistical Analysis**

Continuous variables were summarized using descriptive statistics (number of participants, mean, standard deviation, and 95% confidence interval). Categorical variables were summarized using frequencies and percentages.

Data from the PREVENT study of eculizumab, the SAkuraStar and SAkuraSky studies of satralizumab, and an observational study of azathioprine (AZA) were used to evaluate the long-term comparative efficacy of INEB. ^3-6^ Data from the observational study on AZA included longitudinal attack history data from disease onset up to initiation of treatment with AZA, and these data were extracted and used as part of the untreated or placebo-treated control group. Attack history data following treatment with AZA were used to model the longitudinal risk of attack while on AZA. Hazard ratios (HRs) for the INEB group vs. the historical comparator groups were estimated using Cox proportional hazards (PHs) regression. Time to NMOSD attack was modeled using parametric and flexible survival (spline) models that were fit to the INEB group and the historical comparator groups. The model was previously described. ^2^ In brief, the model compared the time to NMOSD attack in participants treated with INEB in the OLP vs. a historical placebo group and a historical IST group from the studies described in **Supplementary Table 1**. Digitized Kaplan-Meier data for the historical comparator groups were used for the comparisons. The time to NMOSD attack in the INEB treatment group vs. historical comparator groups was estimated using models fit to the Kaplan-Meier data. Diagnostics used for modeling included Akaike’s or Bayesian Information Criteria, visual fit inspection, estimated attack-free survival at 4 years, and clinical validation informed model selection. The appropriate modeling technique was established using PHs and accelerated failure time assumptions. A spline model was used to compare the efficacy of INEB vs. historical placebo and historical ISTs as the time-varying spline with two internal knots, and a normal linear predictor provided the best fit for the data. In the absence of long-term placebo or IST comparator data in the OLP, these synthetic historical comparator groups were used to evaluate the long-term efficacy of INEB. Although the N-MOmentum RCP included a placebo control group, all participants in the OLP of N-MOmentum eventually switched to INEB treatment.

**SUPPLEMENTARY FIGURES**


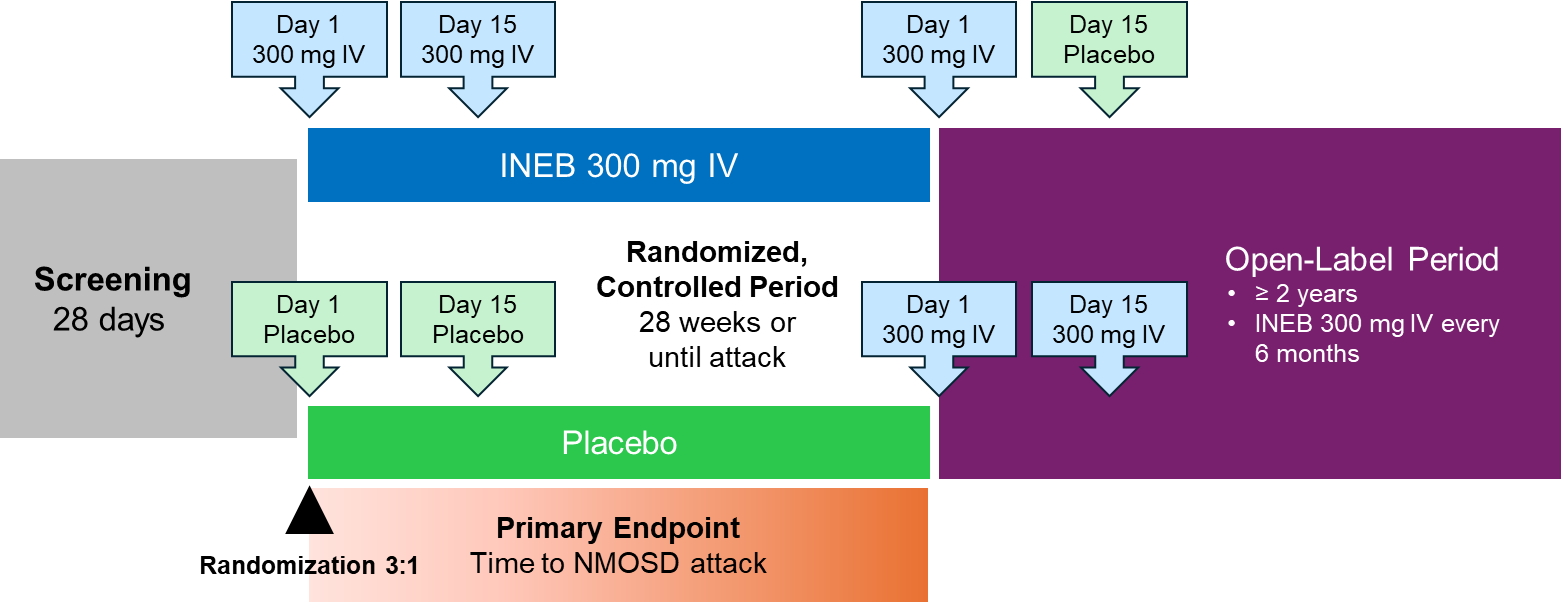


**Supplementary Figure 1.** N-MOmentum trial design.

INEB, inebilizumab; IV, intravenous; NMOSD, neuromyelitis optica spectrum disorder.

**SUPPLEMENTARY TABLES**

**Supplementary Table 1.** NMO-related inpatient hospitalization rates among participants treated with INEB during the N-MOmentum trial.

|  | **AQP4^+^** | |
| --- | --- | --- |
|  | **Prior IST treatment^a^**  **(n=94)** | **IST naïve^b^**  **(n=103)** |
| **Number of NMO-related hospitalizations, n** | 40 | 40 |
| **Total person-years** | 300.35 | 335.7 |
| **Annualized hospitalization rate (95% CI)** | 0.151 (0.084, 0.270) | 0.120 (0.064, 0.223) |

AQP4^+^, aquaporin-4 seropositive; AZA, azathioprine; CI, confidence interval; INEB, inebilizumab; IST, immunosuppressant; MMF, mycophenolate mofetil; NMO, neuromyelitis optica; RTX, rituximab.

^a^Prior IST treatment included AZA, MMF, and/or methotrexate; prior RTX users were excluded.

^b^The IST-naïve group includes some participants who had prior treatment with less commonly used ISTs.

**Supplementary Table 2.** Summary of studies informing the HCGs.

| **HCG** | **Study** | **Study type** | **Treatments** | **n** | **AQP4-IgG status** |
| --- | --- | --- | --- | --- | --- |
| **Placebo**  **(n=106)** | Costanzi^3^ | Retrospective medical record review | Data prior to AZA treatment^a^ | 70 | Mix of seropositive and seronegative |
|  | Pittock^4^  (PREVENT) | RCT | Placebo | 13 | Seropositive |
|  | Traboulsee^5^  (SAkuraStar) | RCT | Placebo | 23 | Seropositive |
| **IST comparators**  **(n=132)** | Costanzi^3^ | Retrospective medical record review | AZA | 70 | Mix of seropositive and seronegative |
|  | Pittock^4^  (PREVENT) | RCT | IST | 34 | Seropositive |
|  | Yamamura^6^  (SAkuraSky) | RCT | IST | 28 | Seropositive |

AQP4-IgG, aquaporin-4–immunoglobulin G; AZA, azathioprine; HCG, historical comparator group; IST, immunosuppressant; RCT, randomized, controlled trial.

^a^The Costanzi et al paper describes treatments prior to AZA treatment, in which 10 patients were treated with ISTs (discontinued 2–12 months prior to AZA treatment) and 31 were treated with immunomodulatory multiple sclerosis therapies (timing not specified).

**SUPPLEMENTAL APPENDIX REFERENCES**

1. B. A. C. Cree, J. L. Bennett, H. J. Kim, et al., "Inebilizumab for the Treatment of Neuromyelitis Optica Spectrum Disorder (N-MOmentum): A Double-Blind, Randomised Placebo-Controlled Phase 2/3 Trial," *Lancet* 394, no. 10206 (2019): 1352-1363.

2. B. A. C. Cree, H. J. Kim, B. G. Weinshenker, et al., "Safety and Efficacy of Inebilizumab for the Treatment of Neuromyelitis Optica Spectrum Disorder: End-of-Study Results From the Open-Label Period of the N-MOmentum Trial," *Lancet Neurology* 23, no. 6 (2024): 588-602.

3. C. Costanzi, M. Matiello, C. F. Lucchinetti, et al., "Azathioprine: Tolerability, Efficacy, and Predictors of Benefit in Neuromyelitis Optica," *Neurology* 77, no. 7 (2011): 659-666.

4. S. J. Pittock, A. Berthele, K. Fujihara, et al., "Eculizumab in Aquaporin-4-Positive Neuromyelitis Optica Spectrum Disorder," *New England Journal of Medicine* 381, no. 7 (2019): 614-625.

5. A. Traboulsee, B. M. Greenberg, J. L. Bennett, et al., "Safety and Efficacy of Satralizumab Monotherapy in Neuromyelitis Optica Spectrum Disorder: A Randomised, Double-Blind, Multicentre, Placebo-Controlled Phase 3 Trial," *Lancet Neurology* 19, no. 5 (2020): 402-412.

6. T. Yamamura, I. Kleiter, K. Fujihara, et al., "Trial of Satralizumab in Neuromyelitis Optica Spectrum Disorder," *New England Journal of Medicine* 381, no. 22 (2019): 2114-2124.
